# Supplementary material for: A secretome atlas of cardiac fibroblasts from healthy and infarcted mouse hearts
Source: Commun Biol. 2025 Apr 29;8:675. doi: 10.1038/s42003-025-08083-y (PMC12041564; doi:10.1038/s42003-025-08083-y)
Supplement: Supplementary file 1 — Supplementary Information [file 42003_2025_8083_MOESM1_ESM.pdf]

Supplementary Information

A secretome atlas of cardiac fibroblasts from healthy and infarcted mouse hearts

Jasmin Bahr[1], Gereon Poschmann[2], Andreas Jungmann[3], Martin Busch[3], Zhaoping Ding[1], Jens Vogt[4], Ria Zalfen[1], Julia Steinhausen[1], Arlen Aurora Euan Martínez[1], Thorsten Wachtmeister[5], Daniel Rickert[5], Tobias Lautwein[5], Christina Alter[1], Junedh M. Amrute[6], Kory J. Lavine[6], Karl Köhrer[5], Bodo Levkau[4,7], Patrick Most[3], Kai Stühler[2,8], Julia Hesse[1,7,*], Jürgen Schrader[1,7,*]

[1]Department of Molecular Cardiology, Medical Faculty and University Hospital Düsseldorf, Heinrich Heine University Düsseldorf, 40225 Düsseldorf, Germany.
[2]Institute for Molecular Medicine, Proteome Research, Medical Faculty and University Hospital Düsseldorf, Heinrich Heine University Düsseldorf, 40225 Düsseldorf, Germany.
[3]Division of Molecular and Translational Cardiology, Department of Internal Medicine III, Heidelberg University Hospital, 69120 Heidelberg, Germany.
[4]Institute of Molecular Medicine III, Medical Faculty and University Hospital Düsseldorf, Heinrich Heine University Düsseldorf, 40225 Düsseldorf, Germany.
[5]Genomics & Transcriptomics Laboratory, Biological and Medical Research Centre (BMFZ), Heinrich Heine University Düsseldorf, 40225 Düsseldorf, Germany.
[6]Center for Cardiovascular Research, Department of Medicine, Cardiovascular Division, Washington University School of Medicine, St. Louis, MO 63110, USA.
[7]CARID, Cardiovascular Research Institute Düsseldorf, Medical Faculty and University Hospital Düsseldorf, Heinrich Heine University Düsseldorf, 40225 Düsseldorf, Germany.
[8]Molecular Proteomics Laboratory, Biological and Medical Research Centre (BMFZ), Heinrich Heine University Düsseldorf, 40225 Düsseldorf, Germany.

*Correspondence:
Dr. Julia Hesse, julia.hesse@uni-duesseldorf.de; Prof. Dr. Jürgen Schrader: schrader@uni-duesseldorf.de

Supplementary Table 1: Reported functions of paracrine/autocrine factors secreted from cCF

Proteins with known functions that were found in the secretome of non-activated CF (cCF) isolated from the mouse hearts 5 days after sham surgery were selected by PubMed search. Proteins are listed in decreasing secretome protein intensity. Quantitative information of the listed proteins can be found in Figure 2 and Supplementary Data 1.

Protein; *gene*	Function	Ref.
**Plasminogen activator inhibitor 1 (PAI-1);** ***Serpine1***	Central function in thrombo-inflammation	1
**Gelsolin (GSN);** ***Gsn***	Important mediator of cardiac fibrosis	2
**Insulin like growth factor binding protein 4 (IGFBP4);** ***Igfbp4***	Cardiogenic growth factor	3
**Pentraxin-related protein 3 (PTX3);** ***Ptx3***	Essential component of the humoral arm of the innate immune system	4
**Complement C3 (C3);** ***C3***	Important component of complement system	5
**Chemokine (C-C motif) ligand 2 (CCL2);** ***Ccl2***	Regulates migration and infiltration of a wide range of immune cells	6
**Pigment epithelium-derived factor (PEDF);** ***Serpinf1***	Potent inhibitor of angiogenesis	7
**Beta-nerve growth factor (NGF);** ***Ngf***	Beneficial actions on cardiomyocytes; angiogenesis	8
**Angiopoietin-like 4 (ANGPTL4);** ***Angptl4***	Antiangiogenic modulatory factor	9
**Proprotein convertase subtilis/kexin 6 (PCSK6);** ***Pcsk6***	Inhibits cardiomyocyte senescence	10
**Follistatin-related protein 1 (FSTL1);** ***Fstl1***	Critical for homeostasis of vascular wall	11
**Superoxide dismutase (SOD3);** ***Sod3***	Redox signaling; modulation of inflammatory response	12
**Tyrosine-protein kinase receptor UFO (AXL);** ***Axl***	Mediates inflammation	13

Supplementary Table 2: Intracellular protein and mRNA transcript levels of proteins of the cCF secretome. The cCF proteome in cell lysates harvested together with the supernatants for the secretome analysis after 8 h of SILAC labeling was assessed by LC-MS/MS (n=4; source data in Supplementary Data 2). Bulk transcriptome analysis with detection of newly synthesized transcripts (thiol-linked alkylation for metabolic sequencing, SLAMseq) was performed in CF isolated from healthy mouse hearts (n=3; source data in Supplementary Data 3). For transcript labeling, CF were incubated for 12 h with 4-thiouridine (S4U). In subsequent sample processing, incorporated S4U in newly synthesized transcripts resulted in T>C conversions that were quantified by sequencing. For selected proteins of the cCF secretome, their protein levels (intensities with SILAC label, Int) in the secretome and proteome, as well as the mRNA transcript levels (counts per million, CPM) and transcript T>C conversion rates are shown. Data are reported as mean ± SD. n.d., not detected.

**Protein/*gene***	**Secretome [Int]**		**Proteome [Int]**		**Transcriptome [CPM]**		**Transcriptome [T>C conversion rate]**	
**PAI1/*Serpine1***	**7.06E+09**	±2.37E+09	**4.75E+06**	±3.11E+06	**340.57**	±180.03	**0.050**	±0.001
**GSN/*Gsn***	**9.31E+08**	±2.49E+08	**n.d.**		**42.81**	±33.62	**0.015**	±0.003
**IGFBP4/*Igfbp4***	**3,81E+08**	±5.13E+07	**n.d.**		**150.94**	±58.19	**0.014**	±0.002
**PTX3/*Ptx3***	**3.51E+08**	±1.04E+08	**3.24E+07**	±2.18E+07	**60.26**	±49.01	**0.045**	±0.003
**C3/*C3***	**1.34E+08**	±6.82E+07	**n.d.**		**51.45**	±25.28	**0.007**	±0.002
**CCL2/*Ccl2***	**8.71E+07**	±3.45E+07	**n.d.**		**36.95**	±33.56	**0.052**	±0.003
**NGF/*Ngf***	**2.11E+07**	±5.77E+06	**n.d.**		**9.58**	±8.36	**n.d.**	
**CTSB/*Ctsb***	**1.83E+07**	±2.42E+07	**2.10E+07**	±4.50E+06	**76.83**	±38.53	**0.011**	±0.003
**CCL9/*Ccl9***	**1.82E+07**	±1.21E+07	**n.d.**		**5.83**	±4.31	**0.028**	±0.006
**IGF1/*Igf1***	**1.57E+07**	±4.92E+06	**n.d.**		**53.84**	±40.86	**0.039**	±0.002
**ANGPTL4/*Angptl4***	**1.50E+07**	±7.18E+06	**n.d.**		**3.912**	±2.35	**0.031**	±0.004
**CFH/*Cfh***	**1.35E+07**	±1.85E+07	**n.d.**		**80.87**	±63.58	**0.015**	±0.000
**PCSK6/*Pcsk6***	**1.30E+07**	±5.56E+06	**n.d.**		**19.82**	±12.56	**0.019**	±0.005
**FSTL1/*Fstl1***	**9.91E+06**	±3.45E+06	**n.d.**		**246.95**	±89.34	**0.030**	±0.000
**LPL/*Lpl***	**3.37E+06**	±5.61E+05	**n.d.**		**148.99**	±85.05	**0.029**	±0.003
**AXL/*Axl***	**2.64E+06**	±1.44E+06	**n.d.**		**21.12**	±10.84	**0.040**	±0.004
**HSP47/*Serpinh1***	**1.84E+06**	±1.12E+06	**1.63E +09**	±5.45E+08	**235.86**	±117.12	**0.022**	±0.002

Supplementary Table 3: Secretome proteins of miCF at day 3 and day 5 post-MI that were significantly changed in comparison to cCF from sham-operated hearts.

Secretome data obtained from miCF isolated 3 days after MI and secretome data obtained from miCF isolated 5 days after MI were matched in terms of significantly upregulated proteins compared to respective sham controls (n=4 each; source data in Supplementary Data 1).

Proteins significantly upregulated				
**only at day 3 post-MI**			**both at day 3 and 5 post-MI**	**only at day 5 post-MI**
AEBP1	FBLN5	NID1	COL1A1	ACTG1
APOE	FBN1	OGN	COL12A1	BMP1
APP	FBN2	PAM	COL16A1	CRLF1
B2M	FRZB	PCOLCE2	CPE	FMOD
C1-INH	FSTL1	PEDF	DNAJB11	IGFBP2
CCDC80	GRN	PLAT	ECM1	PLOD1
CCL9	HSPG2	PLOD3	FN1	SEMA7A
COL1A2	IGF1	PSAP	GDF6	TIMP3
COL3A1	IGFBP7	QSOX1	INHBA	
COL4A1	LAMC1	RNASE4	LAMA2	
COL4A5	LGALS3BP	SERPINA3N	LAMA4	
COL5A2	LGMN	SLIT3	LOX	
COL8A1	LOXL2	SPARC	LOXL3	
CTSA	LTBP3	SRPX2	LTBP2	
CTSB	LTBP4	SVEP1	MATN2	
CTSL	METRNL	TCN2	POSTN	
DAG1	MFAP5	TGFBI	PXDN	
EFEMP1	MFGE8	TNC	SFRP1	
EFEMP2	MGP	WISP2	SLIT2	
FBLN1	MMP2		OPG	

Supplementary Table 4: Secretome proteins of cultured miCF and POSTN$^+$ CF in-vivo 5 days post-MI. Identified proteins in the secretome of miCF isolated 5 days after MI (n=4; source data in Supplementary Data 1) and in the in-vivo-secretome of POSTN$^+$ CF collected in the coronary effluent at day 5 post-MI (n=6; source data in Supplementary Data 4) were matched. Proteins that were significantly enriched in the effluent of AAV9-POSTN-ER-TurboID-transduced mice in comparison to non-transduced control mice 5 days post-MI (n=6; source data in Supplementary Data 4) are marked with an asterisk.

Identified proteins detected						
only in cultured miCF			both in cultured miCF and coronary effluent	only in coronary effluent		
ACTG1	EFEMP2	OPG	APOE*	A1AT2*	EGFR*	ITIH2
ADAM12	EMILIN1	PAI-1	BGN	A1AT3*	EIF4A1*	ITIH3
ADAM15	FBN1	PAM	C1-INH*	A1AT4*	EPHX2	ITIH4*
ADAMTS1	FMOD	PCOLCE2	C3*	A1AT5*	EZR	KLKB1*
ADAMTS2	FNDC1	PCSK6	CFH*	A2AP*	F11*	KNG1*
ADAMTSL3	FRZB	PLAT	COL3A1	ACTA2	F12*	KNG2*
ADM	FSTL1	PLAU	CP*	ACTB	F13B*	KRT36
AEBP1	GAS6	PLOD1	DCN	ACTN2	F2*	KRT76
ANGPTL4	GDF6	PLOD3	FBLN1	AFM*	FABP3	LDHA
APP	GDN	PRELP	FBLN2*	AGT*	FABP4	LDHB
ASPN	HSP47	PROS1	FBLN5	AI182371*	FETUB*	LIFR*
AXL	IGF1	PSAP	FN1	ALB*	FGA	LRG1*
B2M	IGFBP2	PTX3	GRN*	ALDOA	FGB	LYZ1
BMP1	IGFBP3	QPCT	GSN	AMBP*	FGG	LYZ2
CCDC80	IGFBP4	RNASE4	HSPG2	ANT3	FHL2	MB
CCL2	IGFBP7	SDF4	LAMA4	ANXA2	GAPDH	MBL1*
CCL9	INHBA	SEMA7A	LAMB1	APOA1*	GC*	MBL2*
CLSTN1	LAMA2	SFRP1	LAMC1	APOA4*	GM20547*	MDH1
COL12A1	LAMB2	SFRP2	LUM	APOH*	GPD1	MST1*
COL16A1	LGALS3BP	SLIT2	NID1	AZGP1*	GPLD1*	MUG1*
COL1A1	LGMN	SLIT3	OGN	BTD*	GPX3	PCCA
COL1A2	LOX	SOD3	PCOLCE	C1S2*	GSTM1	PCX
COL4A1	LOXL1	SPARC	PEDF*	C4B*	H2AC20	PGAM2
COL4A2	LOXL2	SPARCL1	POSTN	C4BPA*	H2BC3	PGM1
COL4A5	LOXL3	SPON2	PXDN	C5*	H2-Q10*	PKM
COL5A2	LPL	SRPX2	QSOX1*	C6	H4C1	PLG*
COL5A3	LTBP1	SVEP1	SERPINA3N*	C8A*	HBA-A1	PPIB
COL6A1	LTBP2	TCN2		C8B*	HBB-B2	PRDX1
COL6A3	LTBP3	TGFBI		C8G*	HBB-BS	PRDX2
COL8A1	LTBP4	THBS1		C9*	HEP2*	PYGM
CPE	MATN2	THBS2		CA2*	HGFAC*	PZP*
CRLF1	METRNL	TIMP1		CES1B*	HP*	SERPINA3K*
CSF1	MFAP5	TIMP2		CES1C*	HPX*	SERPINA3M
CST3	MFGE8	TIMP3		CFHR1*	HRG*	TF*
CTGF	MGP	TNC		CFHR4*	HSP90AA1	TTR
CTSA	MMP2	TSKU		CFI	HSP90AB1	TUBA1B
CTSB	MMP3	WISP2		CKM	HSPA8	TUBB4B
CTSL	NGF	ZFP462		CLU	ICA*	UBC
DAG1	NID2			COL15A1	IGHG1	VCP
DNAH7C	NTN4			CPB2*	IGHG2C	VIM
DNAJB11	NUCB1			CPG*	IGHM	VTN
DPT	NUCB2			CSRP3	IGKC	1700009N14RIK
ECM1	OAF			EEF1A1	IL1RAP*	
EFEMP1	OLFML3			EEF2	ITIH1*	

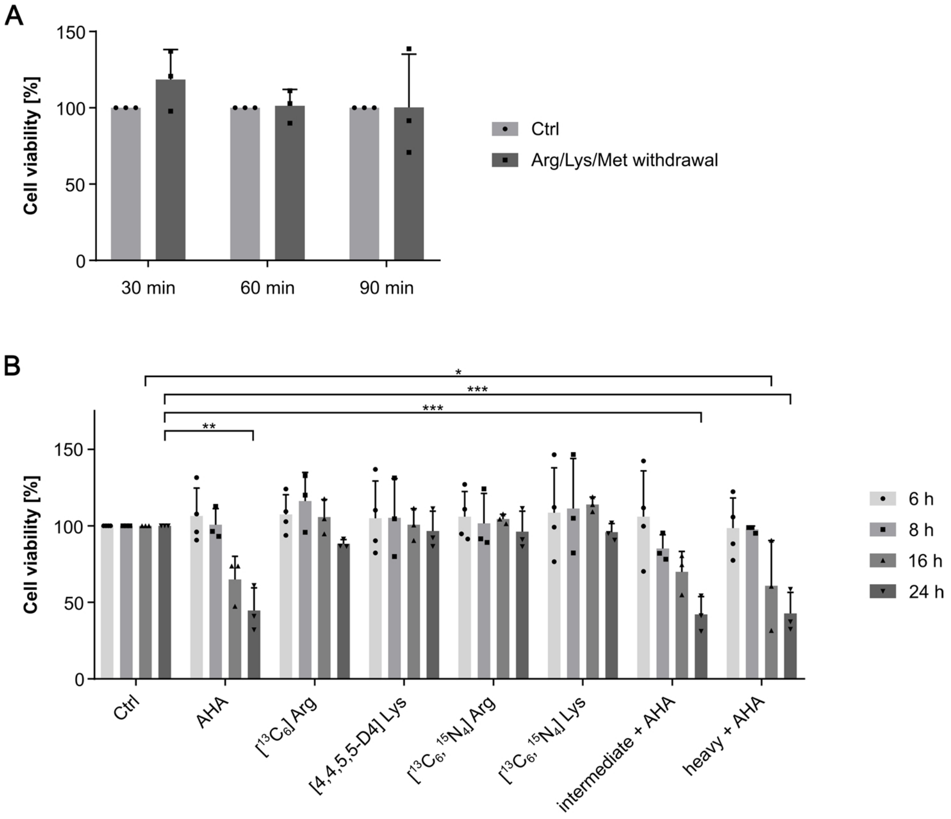

Supplementary Figure 1: Effects of AHA and Arg/Lys isotype labeling on CF cell viability.
A) CF isolated from healthy mouse hearts were incubated in conventional cell culture medium (Ctrl) and depletion medium without L-methionine (Met), L-arginine (Arg), and L-lysine (Lys) (Arg/Lys/Met withdrawal). **B)** After incubation in depletion medium without Met, Arg, and Lys for 1 h, CF were incubated in depletion medium supplemented with AHA, intermediate isotypes [$^{13}C_6$] Arg and [4,4,5,5-D_4] Lys, heavy isotypes [$^{13}C_6$,$^{15}N_4$] Arg and [$^{13}C_6$,$^{15}N_2$] Lys, or combinations of AHA with intermediate and heavy isotypes for the indicated time points. As control (Ctrl), CF were incubated in conventional cell culture medium. All media contained 10% FBS. At the indicated time points, CF cell viability was quantified via Cell Counting Kit-8. The viability of Ctrl cells was set to 100%. Data are shown as mean ± SD (n=3 CF preparations, Ctrl 6 h: n=4 CF preparations; source data in Supplementary Data 5). Two-way ANOVA followed by Dunnett's multiple comparisons test, *P<0.05, **P<0.01, ***P<0.001.

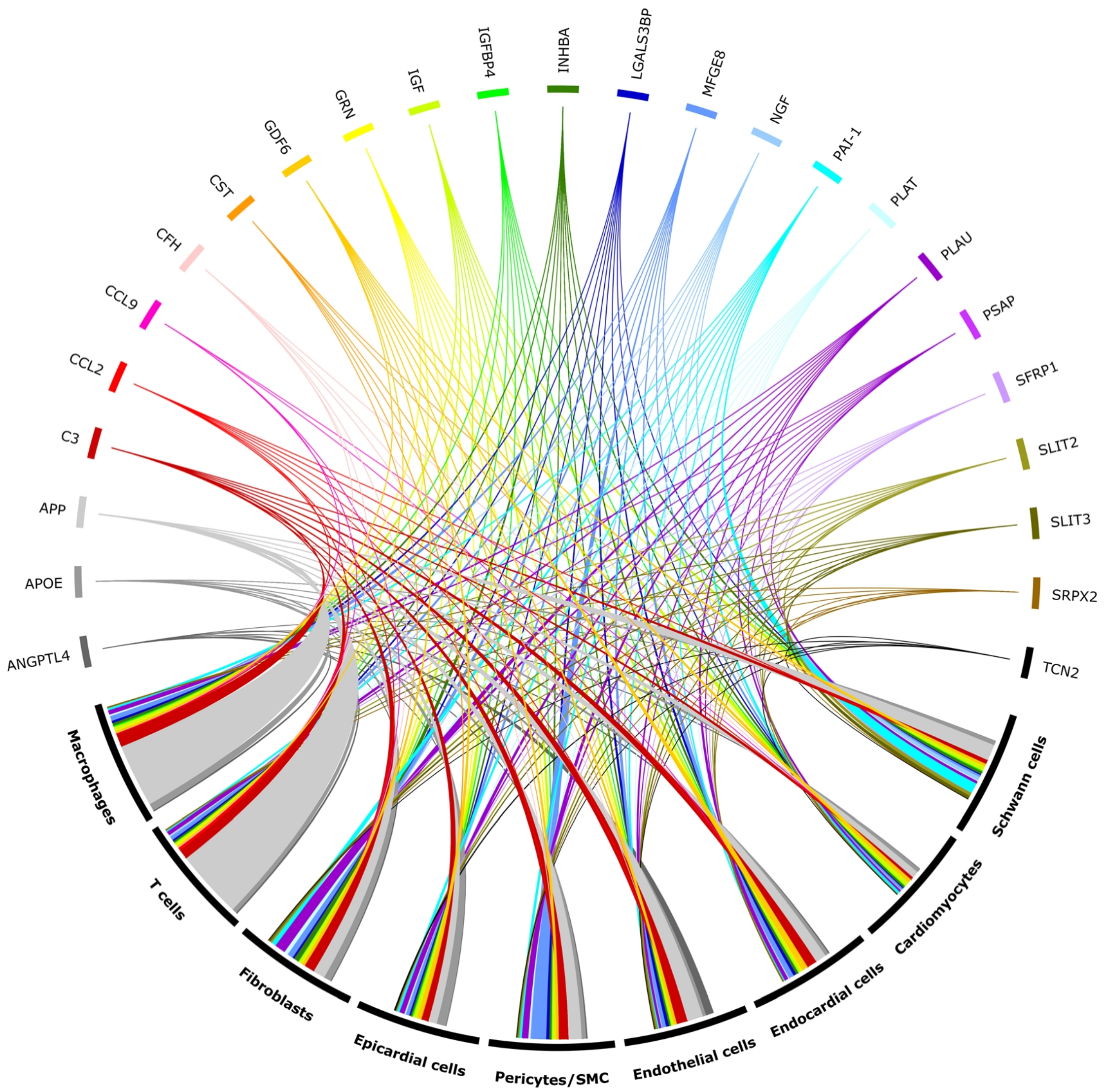

Supplementary Figure 2: Potential cell-cell-communication mediated by cCF paracrine/autocrine factors. Receptors of cCF paracrine/autocrine factors were selected from the ligand-receptor database of CellTalkDB[14] and snRNAseq data from n=1 healthy mouse heart published by Vidal *et al.*[15] (sample Y1, 3,790 cells) were analyzed for their expression. The Circos[16] plot visualizes potential interactions between cCF paracrine factors and their receptor(s) in the different cardiac cell types. The thickness of the lines indicates the receptor expression levels in the respective cell types. Cell types are ordered according to population size.

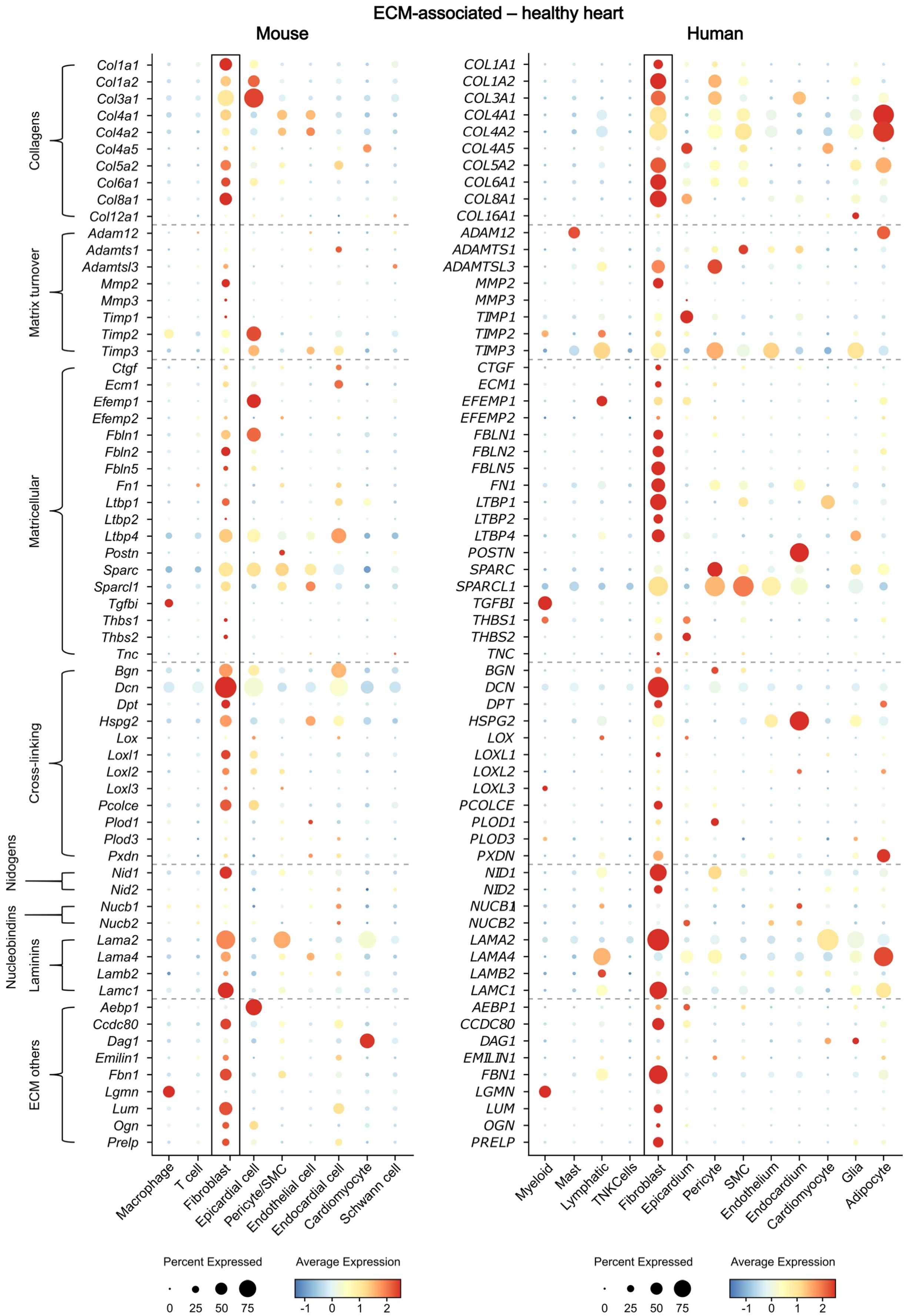
ECM-associated – healthy heart
Mouse
Human
Collagens
Matrix turnover
Matricellular
Cross-linking
Nidogens
Nucleobindins
Laminins
ECM others
Col1a1
Col1a2
Col3a1
Col4a1
Col4a2
Col4a5
Col5a2
Col6a1
Col8a1
Col12a1
Adam12
Adamts1
Adamtsl3
Mmp2
Mmp3
Timp1
Timp2
Timp3
Ctgf
Ecm1
Efemp1
Efemp2
Fbln1
Fbln2
Fbln5
Fn1
Ltbp1
Ltbp2
Ltbp4
Postn
Sparc
Sparcl1
Tgfbi
Thbs1
Thbs2
Tnc
Bgn
Dcn
Dpt
Hspg2
Lox
Loxl1
Loxl2
Loxl3
Pcolce
Plod1
Plod3
Pxdn
Nid1
Nid2
Nucb1
Nucb2
Lama2
Lama4
Lamb2
Lamc1
Aebp1
Ccdc80
Dag1
Emilin1
Fbn1
Lgmn
Lum
Ogn
Prelp
Macrophage
T cell
Fibroblast
Epicardial cell
Pericyte/SMC
Endothelial cell
Endocardial cell
Cardiomyocyte
Schwann cell
COL1A1
COL1A2
COL3A1
COL4A1
COL4A2
COL4A5
COL5A2
COL6A1
COL8A1
COL16A1
ADAM12
ADAMTS1
ADAMTSL3
MMP2
MMP3
TIMP1
TIMP2
TIMP3
CTGF
ECM1
EFEMP1
EFEMP2
FBLN1
FBLN2
FBLN5
FN1
LTBP1
LTBP2
LTBP4
POSTN
SPARC
SPARCL1
TGFBI
THBS1
THBS2
TNC
BGN
DCN
DPT
HSPG2
LOX
LOXL1
LOXL2
LOXL3
PCOLCE
PLOD1
PLOD3
PXDN
NID1
NID2
NUCB1
NUCB2
LAMA2
LAMA4
LAMB2
LAMC1
AEBP1
CCDC80
DAG1
EMILIN1
FBN1
LGMN
LUM
OGN
PRELP
Myeloid
Mast
Lymphatic
TNKCells
Fibroblast
Epicardium
Pericyte
SMC
Endothelium
Endocardium
Cardiomyocyte
Glia
Adipocyte
Percent Expressed
0
25
50
75
Average Expression
-1
0
1
2
Percent Expressed
0
25
50
75
Average Expression
-1
0
1
2

Supplementary Figure 3: Gene expression of ECM-associated proteins identified in secretome analysis of cCF within the major cardiac cell populations as assessed by single-cell transcriptomics.
SnRNAseq data of n=1 healthy mouse heart published by Vidal *et al.*[15] (sample Y1, 3,790 cells; left panel) and snRNAseq data from n=25 human hearts of healthy donors[17–19] (right panel) were re-analyzed. Cellular distribution of gene expression of ECM-associated cCF secretome proteins is visualized as dot plot.

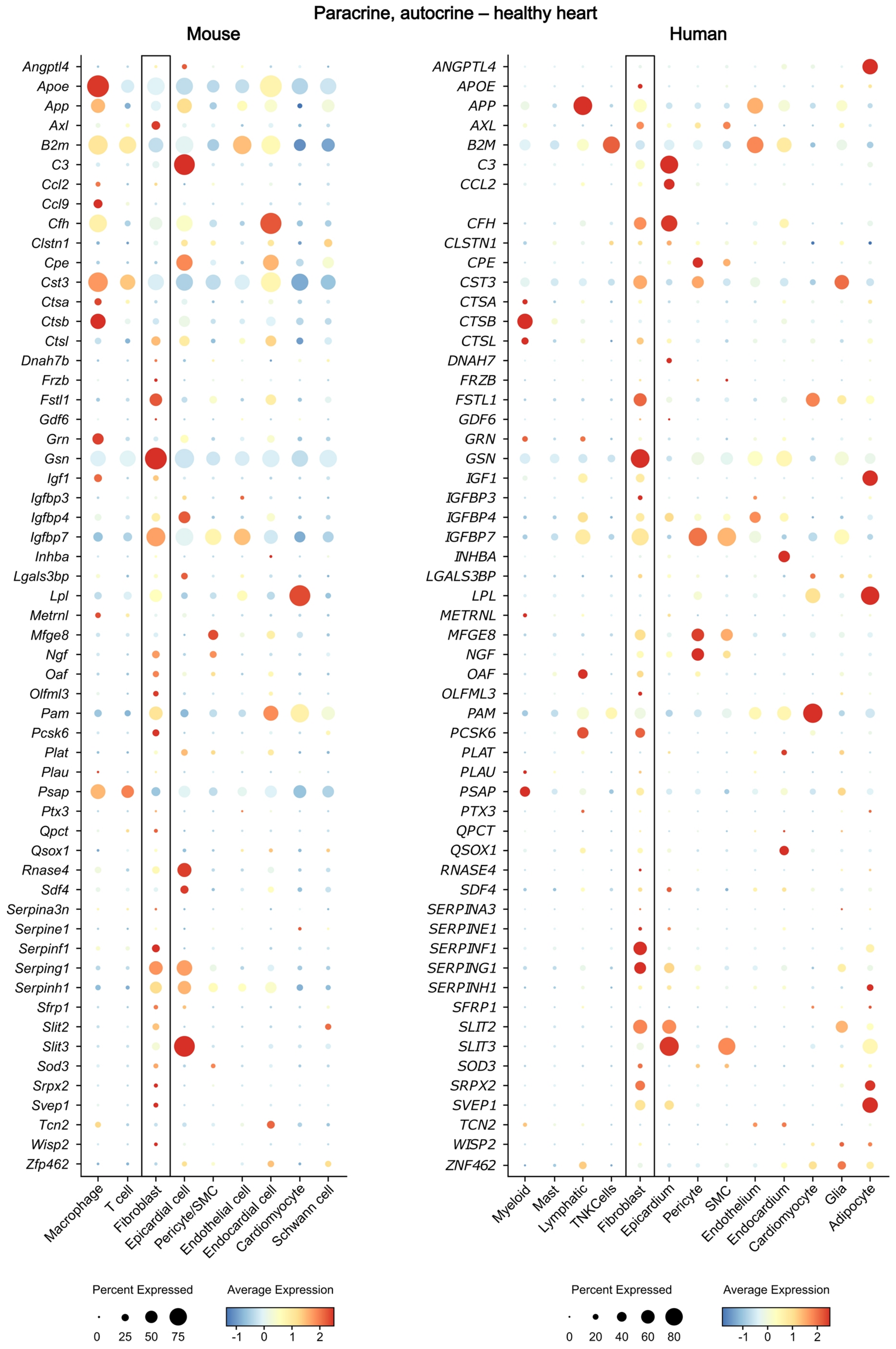

Paracrine, autocrine – healthy heart
Mouse
Human
Angptl4
Apoe
App
Axl
B2m
C3
Ccl2
Ccl9
Cfh
Clstn1
Cpe
Cst3
Ctsa
Ctsb
Ctsl
Dnah7b
Frzb
Fstl1
Gdf6
Grn
Gsn
Igf1
Igfbp3
Igfbp4
Igfbp7
Inhba
Lgals3bp
Lpl
Metrnl
Mfge8
Ngf
Oaf
Olfml3
Pam
Pcsk6
Plat
Plau
Psap
Ptx3
Qpct
Qsox1
Rnase4
Sdf4
Serpina3n
Serpine1
Serpinf1
Serping1
Serpinh1
Sfrp1
Slit2
Slit3
Sod3
Srpx2
Svep1
Tcn2
Wisp2
Zfp462
Macrophage
T cell
Fibroblast
Epicardial cell
Pericyte/SMC
Endothelial cell
Endocardial cell
Cardiomyocyte
Schwann cell
ANGPTL4
APOE
APP
AXL
B2M
C3
CCL2
CFH
CLSTN1
CPE
CST3
CTSA
CTSB
CTSL
DNAH7
FRZB
FSTL1
GDF6
GRN
GSN
IGF1
IGFBP3
IGFBP4
IGFBP7
INHBA
LGALS3BP
LPL
METRNL
MFGE8
NGF
OAF
OLFML3
PAM
PCSK6
PLAT
PLAU
PSAP
PTX3
QPCT
QSOX1
RNASE4
SDF4
SERPINA3
SERPINE1
SERPINF1
SERPING1
SERPINH1
SFRP1
SLIT2
SLIT3
SOD3
SRPX2
SVEP1
TCN2
WISP2
ZNF462
Myeloid
Mast
Lymphatic
TNKCells
Fibroblast
Epicardium
Pericyte
SMC
Endothelium
Endocardium
Cardiomyocyte
Glia
Adipocyte
Percent Expressed
0
25
50
75
Average Expression
-1
0
1
2
Percent Expressed
0
20
40
60
80
Average Expression
-1
0
1
2

Supplementary Figure 4: Gene expression of paracrine/autocrine factors identified in secretome analysis of cCF within the major cardiac cell populations as assessed by single-cell transcriptomics.
SnRNAseq data of n=1 healthy mouse heart published by Vidal et al.[15] (sample Y1, 3,790 cells; left panel) and snRNAseq data from n=25 human hearts of healthy donors[17–19] (right panel) were re-analyzed. Cellular distribution of gene expression of paracrine/autocrine cCF secretome proteins is visualized as dot plot.

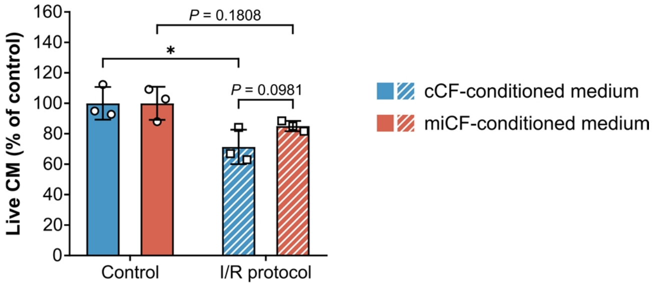

Supplementary Figure 5: Effects of factors secreted by miCF on the viability of cardiomyocytes stressed by hypoxia.

Murine primary adult cardiomyocytes (CM) were isolated from a healthy mouse heart and exposed to hypoxia (1% O_2) for 90 min, followed by incubation for 18 h at standard cell culture conditions (20% O_2), resembling an ischemia/reperfusion (I/R) protocol. During the 18 h ‘reperfusion’ period, medium conditioned for 24 h by cCF (isolated from healthy mouse hearts, n=3 cell preparations) or miCF (isolated 5 days post-MI, n=3 cell preparations) was applied to the CM. As unstressed control, CM were kept at standard cell culture conditions (20% O_2) continuously and were treated with conditioned media in parallel. After treatment, cell viability was assessed by Trypan Blue staining and live CM were quantified. Values were normalized to the unstressed control. Data are shown as mean ± SD (n=3; source data in Supplementary Data 6). Two-way ANOVA followed by Šídák’s multiple comparisons test, *P<0.05.

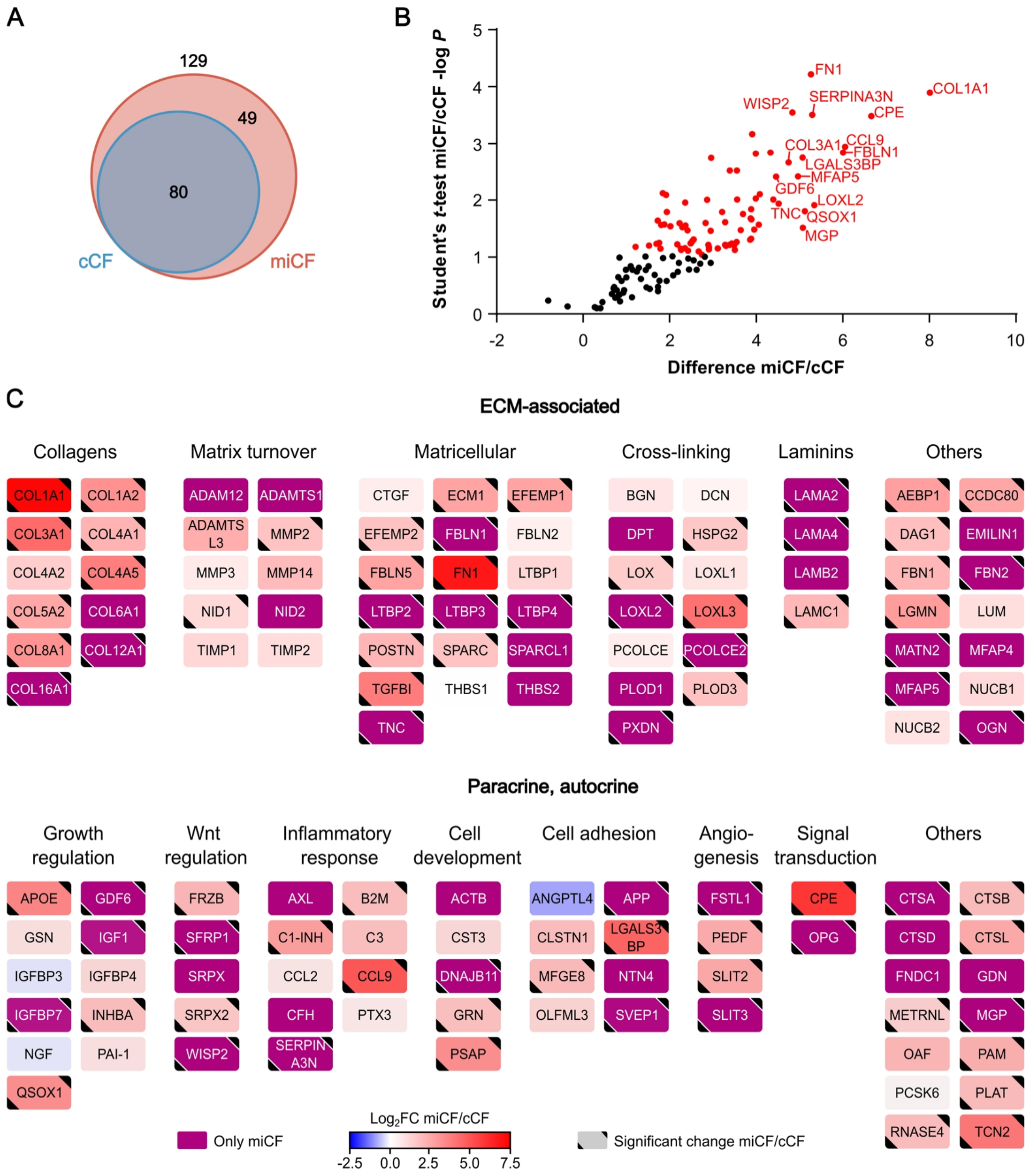

Supplementary Figure 6: MI-induced changes of protein intensities 3 days after infarction.

LC-MS/MS analysis identified 129 secreted proteins from post-MI CF (miCF) isolated from mouse hearts 3 days after I/R surgery (n=4; source data in Supplementary Data 1). These data were compared to the basal secretome data (80 proteins) of cCF isolated from sham-operated hearts 3 days after surgery (n=4; source data in Supplementary Data 1). **A)** Venn diagram[20] of identified proteins. **B)** Volcano plot of differentially secreted proteins. Proteins with significantly different intensities in cCF and miCF samples (Student's *t*-test-based SAM analysis, 5% FDR, S_0=0.1) are highlighted in red (79 proteins). For statistical significance analysis of secreted proteins which were only detected in miCF, an imputation approach of missing base values was performed, using values taken from a downshifted normal distribution (details in Methods). Names of top 15 proteins with highest difference between cCF and miCF are annotated. The shown difference refers to the difference of group mean values of $\log_2$ transformed intensities. **C)** Log2 fold changes (FC) of protein intensities between miCF and cCF samples visualized with Cytoscape[21]. Proteins were grouped in subcategories ECM-associated proteins and paracrine/autocrine factors.

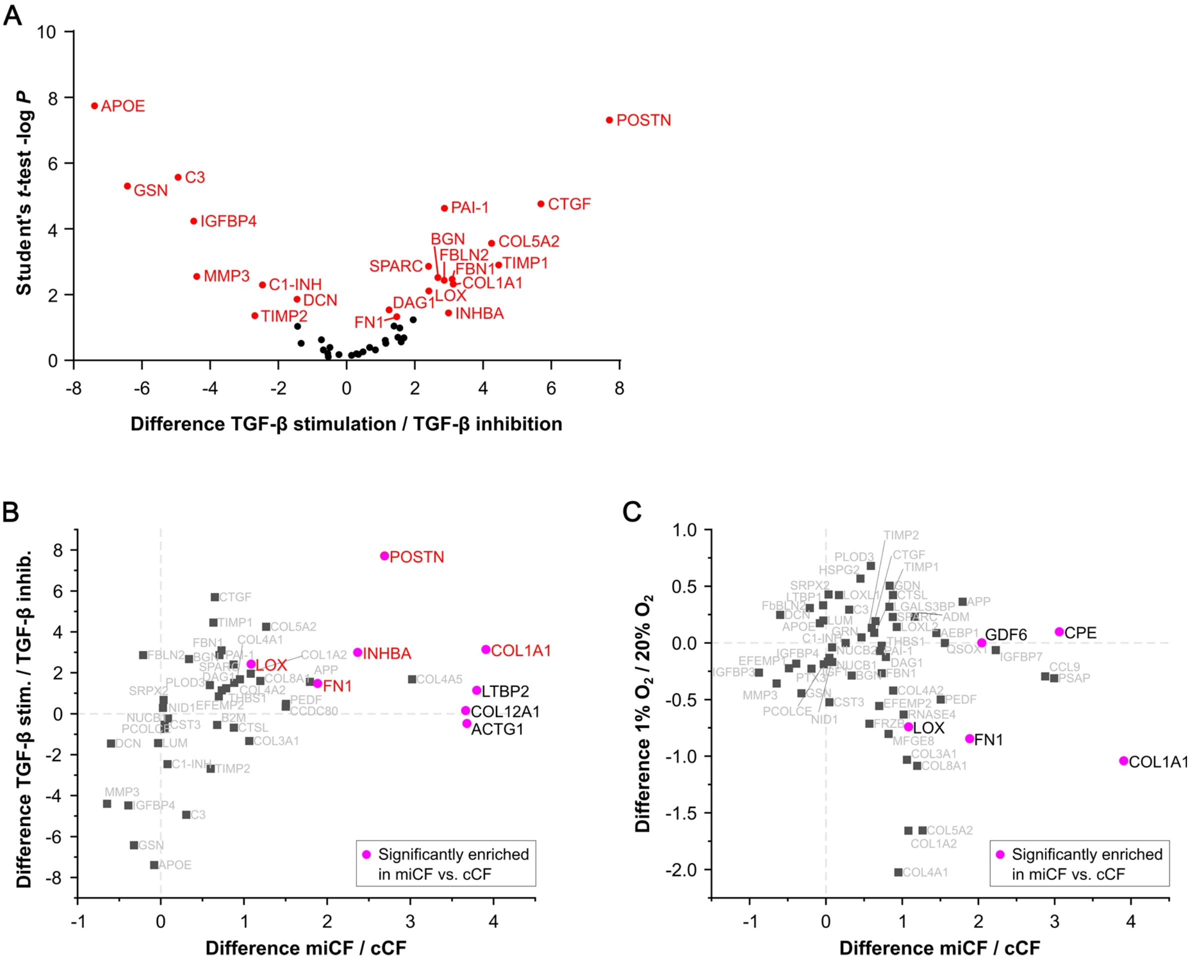

Supplementary Figure 7: Effects of TGF-β stimulation or exposure to hypoxia on the secretome of CF.
A-B) CF isolated from healthy mouse hearts were incubated for 48 h with TGF-β1 (10 ng/ml) or the TGF-β receptor inhibitor SB-431542 (10 µM) as control (n=6 each). Proteins secreted into the cell supernatants were analyzed by LC-MS/MS (source data in Supplementary Data 1). **A)** Volcano plot showing proteins with significantly different intensities (Student's *t*-test-based SAM analysis, 5% FDR, S_0=0.1) in TGF-β-treated samples vs. TGF-β inhibitor controls highlighted in red (22 proteins). Shown are the differences of group mean values of $\log_2$ transformed intensities. **B)** Differences in levels of secreted proteins between TGF-β1-treated CF and controls in comparison to differences between miCF 5 days post-MI and cCF detected in prior experiments (see Figure 4). Only proteins identified in both secretome data sets are shown. Proteins significantly enriched both in TGF-β1-treated CF vs. controls and miCF vs. cCF are highlighted in red. **C)** CF isolated from healthy mouse hearts were exposed to hypoxia (1% O_2) or were incubated under standard cell culture conditions (20% O_2) as control for 8 h (n=6 each). Proteins secreted into the cell supernatants were analyzed by LC-MS/MS. Shown are the differences of secreted proteins between hypoxic CF and controls (source data in Supplementary Data 1) in comparison to differences between miCF isolated 5 days post-MI and cCF detected in prior experiments (see Figure 4).

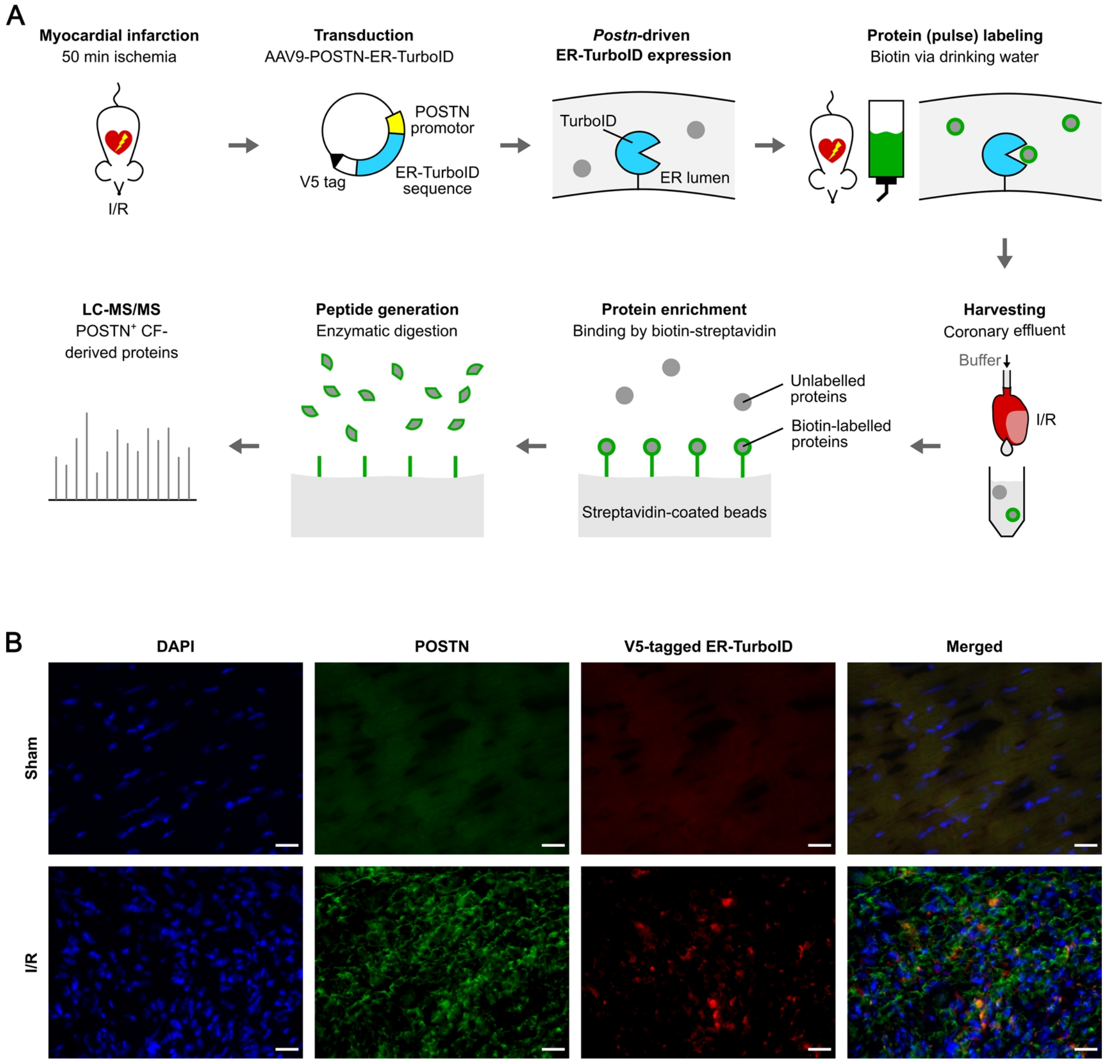

Supplementary Figure 8: Workflow of in-vivo-secretome analysis of POSTN+ CF.
A) At day 1 post MI (50 min ischemia/reperfusion, IR), adeno-associated virus serotype 9 (AAV9) carrying an expression vector encoding V5-tagged ER-localized biotin ligase TurboID (ER-TurboID) under control of the POSTN promotor (AAV9-POSTN-ER-TurboID) was injected i.v.. To allow TurboID expressed in MI-activated POSTN+ CF to biotinylate proximate proteins passing through the ER secretory pathway, biotin was provided via the drinking water for 3 consecutive days before protein harvest. At 5 days post-MI, secreted cardiac proteins were collected in the coronary effluent during Langendorff-based retrograde perfusion. POSTN+ CF-derived, biotin-labelled proteins were bound to streptavidin-coated beads. After stringent washing to remove non-bound proteins, remaining proteins were enzymatically digested and eluted peptides were applied to LC-MS/MS. **B)** Expression of POSTN and V5-tagged ER-TurboID as assessed by immunofluorescence analysis. AAV9-POSTN-ER-TurboID was injected 2 days after MI or sham surgery (n=1 each). Biotin was provided for 3 consecutive days before sacrificing the mice at day 7 post-MI and preparing cryosections. Immunofluorescence analysis was performed with antibodies specific for POSTN and the V5-tag of ER-TurboID. DAPI was used to label nuclei. Representative images of the infarct border zone are shown. Scale bar 20 µm.

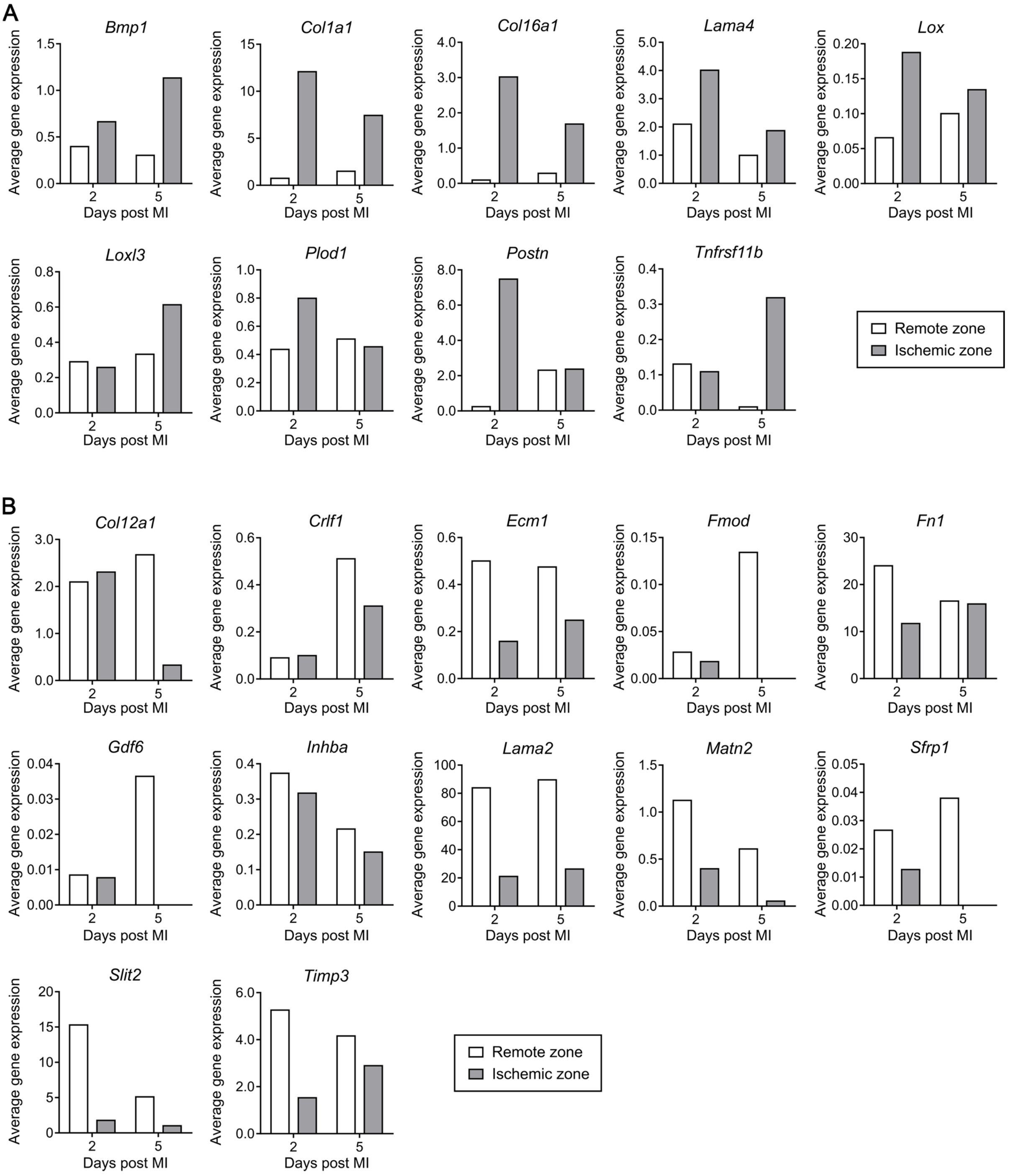

Supplementary Figure 9: Location-dependent differences in expression of miCF-secreted proteins in the human post-MI heart.

Transcript levels of selected significantly changed proteins between the miCF secretome and the cCF secretome in the transcriptome of CF from human heart specimen of the remote zone (RZ) or the ischemic zone (IZ) from patients 2 days (n=2; RZ: 6,260 cells, IZ: 3,009 cells) and 5 days (n=1; RZ: 1,280 cells, IZ: 188 cells) post-MI. Previously published snRNAseq data of a spatial multi-omic map of human MI[22] were reanalyzed. Shown are average expression levels, subdivided in **A)** proteins, which seemed to be prevalently expressed in CF located in the IZ, and **B)** proteins, which seemed to be prevalently expressed in CF located in the RZ.

Supplementary References

1. Morrow, G. B., Whyte, C. S. & Mutch, N. J. A Serpin With a Finger in Many PAIs: PAI-1's Central Function in Thromboinflammation and Cardiovascular Disease. *Front. Cardiovasc. Med.* **8**, 653655 (2021).
2. Jana, S. *et al.* Gelsolin is an important mediator of Angiotensin II-induced activation of cardiac fibroblasts and fibrosis. *FASEB J. Off. Publ. Fed. Am. Soc. Exp. Biol.* **35**, e21932 (2021).
3. Zhu, W. *et al.* IGFBP-4 is an inhibitor of canonical Wnt signalling required for cardiogenesis. *Nature* **454**, 345–349 (2008).
4. Chiari, D. *et al.* The crossroad between autoimmune disorder, tissue remodeling and cancer of the thyroid: The long pentraxin 3 (PTX3). *Front. Endocrinol.* **14**, 1146017 (2023).
5. Erdei, A. *et al.* The versatile functions of complement C3-derived ligands. *Immunol. Rev.* **274**, 127–140 (2016).
6. Zhang, H. *et al.* Role of the CCL2-CCR2 axis in cardiovascular disease: Pathogenesis and clinical implications. *Front. Immunol.* **13**, 975367 (2022).
7. Zhang, H. *et al.* PEDF and 34-mer inhibit angiogenesis in the heart by inducing tip cells apoptosis via up-regulating PPAR-γ to increase surface FasL. *Apoptosis Int. J. Program. Cell Death* **21**, 60–68 (2016).
8. Pius-Sadowska, E. & Machaliński, B. Pleiotropic activity of nerve growth factor in regulating cardiac functions and counteracting pathogenesis. *ESC Heart Fail.* **8**, 974–987 (2021).
9. Ito, Y. *et al.* Inhibition of angiogenesis and vascular leakiness by angiopoietin-related protein 4. *Cancer Res.* **63**, 6651–6657 (2003).
10. Zhan, W. *et al.* Pcsk6 Deficiency Promotes Cardiomyocyte Senescence by Modulating Ddit3-Mediated ER Stress. *Genes* **13**, 711 (2022).
11. Jiang, H. *et al.* Angiocrine FSTL1 (Follistatin-Like Protein 1) Insufficiency Leads to Atrial and Venous Wall Fibrosis via SMAD3 Activation. *Arterioscler. Thromb. Vasc. Biol.* **40**, 958–972 (2020).
12. Hu, L., Zachariae, E. D., Larsen, U. G., Vilhardt, F. & Petersen, S. V. The dynamic uptake and release of SOD3 from intracellular stores in macrophages modulates the inflammatory response. *Redox Biol.* **26**, 101268 (2019).
13. Chen, W. *et al.* Role of Axl in target organ inflammation and damage due to hypertensive aortic remodeling. *Am. J. Physiol. Heart Circ. Physiol.* **323**, H917–H933 (2022).
14. Shao, X. *et al.* CellTalkDB: a manually curated database of ligand-receptor interactions in humans and mice. *Brief. Bioinform.* **22**, bbaa269 (2021).
15. Vidal, R. *et al.* Transcriptional heterogeneity of fibroblasts is a hallmark of the aging heart. *JCI Insight* **4**, (2019).
16. Krzywinski, M. *et al.* Circos: An information aesthetic for comparative genomics. *Genome Res.* **19**, 1639–1645 (2009).
17. Amrute, J. M. *et al.* Defining cardiac functional recovery in end-stage heart failure at single-cell resolution. *Nat. Cardiovasc. Res.* **2**, 399–416 (2023).
18. Koenig, A. L. *et al.* Single-cell transcriptomics reveals cell-type-specific diversification in human heart failure. *Nat. Cardiovasc. Res.* **1**, 263–280 (2022).
19. Amrute, J. M. *et al.* Targeting immune–fibroblast cell communication in heart failure. *Nature* **635**, 423–433 (2024).
20. Hulsen, T. DeepVenn -- a web application for the creation of area-proportional Venn diagrams using the deep learning framework Tensorflow.js. Preprint at https://doi.org/10.48550/arXiv.2210.04597 (2022).
21. Shannon, P. *et al.* Cytoscape: A Software Environment for Integrated Models of Biomolecular Interaction Networks. *Genome Res.* **13**, 2498–2504 (2003).
22. Kuppe, C. *et al.* Spatial multi-omic map of human myocardial infarction. *Nature* **608**, 766–777 (2022).
