# Supplementary material for: A secretome atlas of cardiac fibroblasts from healthy and infarcted mouse hearts
Source: Commun Biol. 2025 Apr 29;8:675. doi: 10.1038/s42003-025-08083-y (PMC12041564; doi:10.1038/s42003-025-08083-y)
Supplement: Supplementary file 8 — Description of Additional Supplementary Materials [file 42003_2025_8083_MOESM8_ESM.docx]

### Description of Additional Supplementary Files

**File name: Supplementary Data 1**

Description: Secretome LC-MS/MS data behind Figures 2, 3, 4, and 6, Supplementary Tables 2, 3, and 4, and Supplementary Figures 6 and 7.

**File name: Supplementary Data 2**

Description: Proteome LC-MS/MS data behind Figures 2E and 3, and Supplementary Table 2.

**File name: Supplementary Data 3**

Description: Transcriptome (SLAMseq) data behind Figure 3, Supplementary Table 2.

**File name: Supplementary Data 4**

Description: In-vivo-secretome LC-MS/MS data behind Figure 7.

**File name: Supplementary Data 5**

Description: CF cell viability assay data behind Supplementary Figure 1.

**File name: Supplementary Data 6**

Description: Cardiomyocyte cell protection assay data behind Supplementary Figure 5.
