# Supplementary material for: A secretome atlas of cardiac fibroblasts from healthy and infarcted mouse hearts
Source: Commun Biol. 2025 Apr 29;8:675. doi: 10.1038/s42003-025-08083-y (PMC12041564; doi:10.1038/s42003-025-08083-y)
Supplement: Supplementary file 9 — Reporting Summary [file 42003_2025_8083_MOESM9_ESM.pdf]

nature portfolio

Corresponding author(s): Julia Hesse, Jürgen Schrader

Last updated by author(s): Mar 28, 2025

Reporting Summary

Nature Portfolio wishes to improve the reproducibility of the work that we publish. This form provides structure for consistency and transparency in reporting. For further information on Nature Portfolio policies, see our Editorial Policies and the Editorial Policy Checklist.

Statistics

For all statistical analyses, confirm that the following items are present in the figure legend, table legend, main text, or Methods section.

n/a	Confirmed	
☐	☒	The exact sample size (*n*) for each experimental group/condition, given as a discrete number and unit of measurement
☐	☒	A statement on whether measurements were taken from distinct samples or whether the same sample was measured repeatedly
☐	☒	The statistical test(s) used AND whether they are one- or two-sided<br>*Only common tests should be described solely by name; describe more complex techniques in the Methods section.*
☒	☐	A description of all covariates tested
☐	☒	A description of any assumptions or corrections, such as tests of normality and adjustment for multiple comparisons
☐	☒	A full description of the statistical parameters including central tendency (e.g. means) or other basic estimates (e.g. regression coefficient) AND variation (e.g. standard deviation) or associated estimates of uncertainty (e.g. confidence intervals)
☒	☐	For null hypothesis testing, the test statistic (e.g. *F*, *t*, *r*) with confidence intervals, effect sizes, degrees of freedom and *P* value noted<br>*Give P values as exact values whenever suitable.*
☒	☐	For Bayesian analysis, information on the choice of priors and Markov chain Monte Carlo settings
☒	☐	For hierarchical and complex designs, identification of the appropriate level for tests and full reporting of outcomes
☒	☐	Estimates of effect sizes (e.g. Cohen's *d*, Pearson's *r*), indicating how they were calculated

Our web collection on statistics for biologists contains articles on many of the points above.

Software and code

Policy information about availability of computer code

Data collection	Mass spectrometry data was collected using a Fusion Lumos mass spectrometer (Thermo Fisher Scientific). NGS library sequencing was performed using the Illumina NextSeq1000/2000 system.
Data analysis	Mass spectrometry data analysis was performed using MaxQuant 2.1.3.0 and 2.0.3.0 (Max Planck Institute for Biochemistry, Planegg, Germany) and Proteome Discoverer 2.4.1.15 (Thermo Fisher Scientific) as described in the Methods section. SLAMseq data were analyzed using the SLAM-DUNK software (https://t-neumann.github.io/slamdunk/). ScRNAseq and snRNAseq data were analyzed using the R Seurat package v3.0 (https://satijalab.org/seurat/). Data were visualized using GraphPad Prism 8 and Microsoft Excel as well as Circos 0.69 (https://circos.ca/), DeepVenn (https://www.deepvenn.com/), and Cytoscape 3.9.1 (https://cytoscape.org/).

For manuscripts utilizing custom algorithms or software that are central to the research but not yet described in published literature, software must be made available to editors and reviewers. We strongly encourage code deposition in a community repository (e.g. GitHub). See the Nature Portfolio guidelines for submitting code & software for further information.

Data

Policy information about availability of data

All manuscripts must include a data availability statement. This statement should provide the following information, where applicable:

- Accession codes, unique identifiers, or web links for publicly available datasets
- A description of any restrictions on data availability
- For clinical datasets or third party data, please ensure that the statement adheres to our policy

Mass spectrometry data have been deposited to the ProteomeXchange Consortium via the PRIDE partner repository with the dataset identifiers PXD046238 (https://www.ebi.ac.uk/pride/archive/projects/PXD046238), PXD053791 (https://www.ebi.ac.uk/pride/archive/projects/PXD053791), and PXD060926 (https://www.ebi.ac.uk/pride/archive/projects/PXD060926). Metabolic RNA sequencing data have been deposited in NCBI's Gene Expression Omnibus and are accessible through GEO Series accession number GSE293090 (https://www.ncbi.nlm.nih.gov/geo/query/acc.cgi?acc=GSE293090). Source data underlying the presented graphs and charts are provided in the Supplementary Data files. All other data supporting the findings of this study are available from the corresponding authors upon reasonable request.

Research involving human participants, their data, or biological material

Policy information about studies with human participants or human data. See also policy information about sex, gender (identity/presentation), and sexual orientation and race, ethnicity and racism.

Reporting on sex and gender	n/a
Reporting on race, ethnicity, or other socially relevant groupings	n/a
Population characteristics	n/a
Recruitment	n/a
Ethics oversight	n/a

Note that full information on the approval of the study protocol must also be provided in the manuscript.

Field-specific reporting

Please select the one below that is the best fit for your research. If you are not sure, read the appropriate sections before making your selection.

☒ Life sciences ☐ Behavioural & social sciences ☐ Ecological, evolutionary & environmental sciences

For a reference copy of the document with all sections, see nature.com/documents/nr-reporting-summary-flat.pdf

Life sciences study design

All studies must disclose on these points even when the disclosure is negative.

Sample size	For in-vitro-secretome analysis, n=4 cardiac fibroblast preparations from post-MI hearts and n=4 cardiac fibroblast preparations from sham hearts (cCF) were used at each time point (3 days and 5 days post surgery). For in-vitro-secretome analysis after in-vitro stimulation, n=6 cardiac fibroblast preparations were used for each group (stimulation vs. control). For the cardiomyocyte cell protection assay, supernatants from n=3 fibroblast preparations from post-MI hearts (miCF) and n=3 fibroblast preparations from sham hearts (cCF) were used. For SLAMseq analysis, n=3 cardiac fibroblast preparations were used. For in-vivo-secretome analysis, coronary effluent was collected from n=6 mice with AAV9-POSTN-ER-TurboID injection and n=6 mice without injection.
Data exclusions	No data were excluded. The procedure of exclusion of peptides/proteins from further analyses is described in the Methods section.
Replication	Experiments were performed with at least n=3 biological replicates. For each experiment, the n numbers are stated in the Figure Legends.
Randomization	No randomization was applied.
Blinding	No blinding was applied.

Reporting for specific materials, systems and methods

We require information from authors about some types of materials, experimental systems and methods used in many studies. Here, indicate whether each material, system or method listed is relevant to your study. If you are not sure if a list item applies to your research, read the appropriate section before selecting a response.

Materials & experimental systems

n/a	Involved in the study
☐	☒ Antibodies
☒	☐ Eukaryotic cell lines
☒	☐ Palaeontology and archaeology
☐	☒ Animals and other organisms
☒	☐ Clinical data
☒	☐ Dual use research of concern
☒	☐ Plants

Methods

n/a	Involved in the study
☒	☐ ChIP-seq
☒	☐ Flow cytometry
☒	☐ MRI-based neuroimaging

Antibodies

Antibodies used

Periostin was detected using rabbit polyclonal anti-periostin antibody (CAT# TA309212; OriGene, Rockville, USA). V5-tagged ER-TurboID was detected using mouse monoclonal anti-V5 tag antibody (clone 2F11F7, CAT# 37-7500 ; Thermo Fisher Scientific, Waltham, USA).

Validation

Anti-periostin antibody:
Immunogen: Synthetic peptide corresponding to amino acid residues from the fasciclin domain 1 of mouse periostin immunogen.
Purification: Affinity purified.
Anti-V5 tag antibody:
Immunogen: sequence -Gly-Lys-Pro-Ile-Pro-Asn-Pro-Leu-Leu-Gly-Leu-Asp-Ser-Thr-
Purification: Protein A.
RRID:AB_2533339

Animals and other research organisms

Policy information about studies involving animals; ARRIVE guidelines recommended for reporting animal research, and Sex and Gender in Research

Laboratory animals

C57Bl/6J wildtype mice at an age of 10-12 weeks and body weight of 20-25 g, purchased from Janvier (Le Genest Isle, France), were used.

Wild animals

The study did not involve wild animals.

Reporting on sex

Only male mice were used.

Field-collected samples

The study did not involve samples collected from the field.

Ethics oversight

We have complied with all relevant ethical regulations for animal use. All experiments were performed according to the institutional and national guidelines for animal care in conformity to directive 2010/63/EU and were approved by the Landesamt für Natur, Umwelt und Verbraucherschutz Nordrhein-Westfalen (reference number 81-02.04.2020.A351).

Note that full information on the approval of the study protocol must also be provided in the manuscript.

Plants

Seed stocks

n/a

Novel plant genotypes

n/a

Authentication

n/a
